# Application of Vibrating Reverse Osmosis Technology for Nutrient Recovery from Pig Slurry in a Circular Economy Model

**DOI:** 10.3390/membranes12090848

**Published:** 2022-08-30

**Authors:** Esther Vega, Lidia Paredes, Evan A. N. Marks, Berta Singla, Omar Castaño-Sánchez, Carme Casas, Rosa Vilaplana, Mabel Mora, Sergio Ponsá, Laia Llenas

**Affiliations:** BETA Technological Center, TECNIO Network, University of Vic—Central University of Catalonia, C/de Roda 70, 08500 Vic, Spain

**Keywords:** agronomic assessment, bio-based fertilizers, digestate, nutrient recovery, pig slurry, reverse osmosis, vibratory shear enhanced processing

## Abstract

The rapid growth of the livestock sector in some areas of Europe has caused an imbalance between the generation of livestock manure and the availability of agricultural soil for its direct application as a fertilizer. Since the transport of pig slurry to other areas with nutrient-deficient soils is costly from an economic point of view due to its high water content, the application of new technologies for the concentration of this waste is considered key for reducing management costs. Consequently, the main objective of this study was to demonstrate the potential of vibratory shear enhanced processing (VSEP) operated with reverse osmosis membranes to recover nutrients from the liquid fractions of pig slurry (LF-pig slurry) and digestate (LF-digestate) and obtain concentrated fertilizing products. Use of the VSEP unit permitted reductions in the water contents of the LF-pig slurry and LF-digestate, around 77% and 67%, respectively. Both VSEP concentrates were characterized by their significant nutrient contents and showed a nitrogen fertilizer replacement value similar to that of mineral fertilizer as demonstrated in a barley crop pot-test, although the salinity of the digestate concentrate was identified as a key limitation, negatively impacting the agronomic yield of the test crop.

## 1. Introduction

Livestock manure and its digested by-products (digestate) are considered valuable bio-based fertilizer resources for agricultural land due to their contents of nutrients and organic matter, facilitating water-holding capacity and improving the productive yield of target crops [1,2]. However, the uncontrolled application of livestock manure has generated serious groundwater pollution by nitrates and the eutrophication of surface water [3,4]. To deal with this problem associated with the dramatic growth of the livestock sector, the European Community established Nitrate Directive guidelines (Council Directive 91/676/EEC) [5] limiting the application of nitrogen in agricultural soils to 170 kg N ha^−1^. Moreover, this restriction has resulted in livestock waste exporting to deficit areas since most of the EU is characterized by nitrogen surpluses, reaching an average surplus of 49–80 Kg N ha^−1^ on agricultural land for the years between 2004 and 2011 [6]. 

In addition, since wastes derived from livestock are increasingly generated at facilities far away from intensive cropping areas demanding fertilizer products, this creates the need to transport them, despite the high costs. In that sense, different management strategies and treatment technologies have been developed and evaluated in recent years to maximize the concentration and reduce the volume of livestock wastes, facilitating their transport to deficit areas [7]. On the other hand, nowadays the implementation of biorefineries integrated with different combinations of technologies for the proper management of livestock manure is considered an important challenge which requires further research in order to boost the livestock sector towards the circular economy model [8,9,10]. 

Pressure-driven membrane technologies such as microfiltration, ultrafiltration, nanofiltration and reverse osmosis (RO) provide a suitable option for recovering both nutrients and reusable water during the treatment of livestock waste streams [11,12]. However, a decline in permeate flux due to the accumulation of substances on the membrane surface or within the membrane pore structure is the main technical limitation of these types of technologies [13]. Backwashing and chemical cleaning procedures reestablish the permeate flux and the efficiency of the process, but this strategy significantly increases operating costs and reduces the lifespan of the membrane modules. Thus, numerous mitigation measures, including the efficient control of membrane fouling by adjustment of the operational conditions of the process, the selection of the optimal pre-treatments and the use of appropriate membranes, are required [14].

Vibratory shear enhanced processing (VSEP) is a membrane-separation technology based on the application of intense shear waves on the membrane surface to reduce the membrane fouling and, in turn, increase the separation efficiency. “Plate and frame” membrane configuration is subjected to torsional vibration in order to generate shear forces on the membrane surface which are higher than those achieved in conventional RO modules [15,16,17].

The feasibility of the VSEP technology has been investigated in different treatment fields such as the pulp and paper mill industries [18], the dairy industry [19,20,21], landfill leachate [22], and desalination [23,24]. So far, very few studies have been carried out on the removal of macronutrients (N, P, K) during manure treatment through VSEP technology. Researchers agree on obtaining high nitrogen removal efficiencies around 96% for livestock waste treatment, whereas these yields decline for phosphorus removal, not exceeding 60% [25,26,27]. It should be highlighted that previous efforts have relied on pre-treatments with biological systems to comply with discharge limits, and VSEP technology for direct livestock waste treatment (with no precursor technologies) has not been evaluated as a unitary treatment system. 

Although different studies can be found in the literature about the application of the VSEP technology, most of them are based on the evaluation of the technology to treat different wastes and recover nutrients, and very little information is available about the agronomic assessment of the obtained concentrated products to be used as bio-based fertilizers in agriculture. In this sense, Vaneeckhaute et al. (2019) [28], during their study focused on evaluating the performance of the VSEP technology in resource recovery from the liquid fraction of digestates, identified the need for evaluating the impact of the concentrate products obtained with the VSEP technology on the plant growth and soil quality in future research. Yoon et al. (2004) [29] investigated the effect of applying the concentrate obtained with the VSEP process on rice crops by means of agronomic tests. The authors state that the concentrate obtained from the treatment of piggery wastewater by means of VSEP systems provided the same yields as a mineral fertilizer. Nevertheless, neither operational conditions nor recovery yields for the VSEP process were evaluated, and it is necessary to investigate these aspects in detail in future experiments. 

The main objective of this work was to assess the performance of the VSEP technology for the treatment of the liquid fractions of pig slurry and digestate in order to recover nutrients and obtain a nutrient-rich concentrate with the potential to be applied as a fertilizing product in agriculture. Moreover, the agronomic value of the obtained concentrate was assessed and compared with a mineral fertilizer by means of a pot-test. 

## 2. Materials and Methods

### 2.1. Selection of Feedstocks

Liquid fractions from digestate (LF-digestate) and pig slurry (LF-pig slurry) were used as feedstocks for VSEP tests. Digestate was collected from an on-farm livestock waste treatment plant where pig slurry, slaughterhouse sludge and food waste are fed into a mesophilic anaerobic digestor with a working volume of 5000 m^3^ and a hydraulic retention time of 75–80 d [30]. The liquid fraction from pig slurry was collected in a fattening pig farm where mechanical solid–liquid separation with a press screw separator is undertaken [31]. Before its treatment with the VSEP system, the liquid fraction from pig slurry was screened using a SWECO vibrating screen provided by New Logic Research, Inc. (Emeryville, CA, USA) with a screen size down to 120 µm in order to remove abrasive solids which could damage the membranes and pumps integrated in the VSEP unit. Additionally, the digestate was pretreated in a centrifuge (without addition of chemical agents such as polyelectrolyte) and the liquid fraction obtained was subsequently treated using the same SWECO vibrating screen before its treatment with the VSEP system.

### 2.2. Vibratory Shear Enhanced Processing System

A VSEP Series LP membrane filtration unit and periphery equipment (Figures S1 and S2) were provided by New Logic Research, Inc. (Emeryville, CA, USA) to demonstrate the potential of the VSEP technology to treat digestate and pig slurry streams. The VSEP LP pilot was equipped with a filter pack of circular flat sheet reverse osmosis ESPA membranes provided by New Logic Research, Inc. (Emeryville, CA, USA) with a total membrane surface area of 1.55 m^2^. The membrane module was subjected to a vibration by means of a torsion spring in order to minimize the fouling of the filtration media.

### 2.3. Vibratory Shear Enhanced Processing Operation

**Figure 1:**
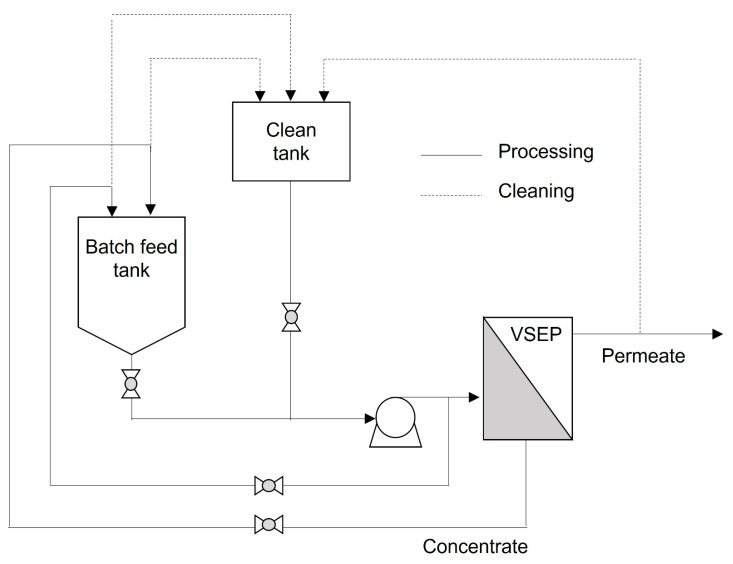
Batch mode pilot testing process flow diagram.

The system was set up in batch mode and the liquid fractions from the digestate and pig slurry were processed through the VSEP LP unit in a series of different batches (initial volume of feedstock between 80 and 100 L). The batch mode pilot testing process flow diagram is shown in Figure 1. In batch mode, a finite volume of feed is processed. The concentrate (or reject) is sent back to the feed tank and the permeate (or filtrate) is sent to a separate tank. As the permeate is removed, the feed tank volume is decreased and becomes concentrated until an end point based on flow or another desired set point. The concentrate flow is held constant to maximize cross flow, and, in combination with vibration, will reduce the overall rate of fouling. The advantages of batch mode are slightly higher flux rates and slightly lower cleaning frequency, at the cost of needing additional tanks and peripheral equipment. 

The system was tested with clean water to obtain the water flux of the membrane module. This value was used as a reference for cleanings. Once the filter pack condition was verified and it was ensured that there were no apparent system leaks, the system was operated with the feedstocks to begin data collection. During the operation of the VSEP system, the membrane module was vibrated with a fixed frequency of 55 Hz and a vibratory amplitude of 1.27 cm.

The working pressure applied during each batch test was determined for each feedstock since this operating parameter depends on its composition. The durability of each batch was conditioned by the permeate flow rate. The permeate flow rate is considered the key parameter for proper operation of the VSEP system, whereas a decrease in the permeate flow implies a significant fouling of the membrane surface [32]. In this sense, the permeate and concentrate flow rates were measured every 10 min during the execution of the batch tests and the VSEP system was operated until permeate flows lower than 7.7 L m^−2^ h^−1^ were achieved.

During the execution of the batch tests the following parameters were monitored: permeate flow rate, concentrate flow rate, inlet pressure to VSEP system, outlet pressure to VSEP system, permeate conductivity, concentrate conductivity and temperature of the liquid product in the feed tank.

Multiple cleaning cycles were carried out to restore the water flux at the end of each batch test. The cleaning agents used, including an acidic liquid cleaner (NLR 404) and an alkaline liquid cleaner (NLR 505), were provided by New Logic Research (Emeryville, CA, USA). A solution of each cleaning product was prepared at 3% (*w*/*w*) and was recirculated through the VSEP unit from the feed tank. Clean-in-place was conducted over 30 min with each cleaner and hot water (40 °C) in order to increase the efficiency of the cleaning method. At the end of the multiple cleanings, a test with clean water was conducted in order to verify that the water flux of the membrane module was restored.

### 2.4. Analytical Methods

Feedstocks (liquid fractions from digestate and pig slurry) and VSEP products (permeate and concentrate) were analyzed in terms of pH, conductivity (EC), total solids (TS), volatile solids (VS), total Kjeldahl nitrogen (TKN), ammonium (N-NH_4_^+^), total phosphorous (TP), potassium (K), copper (Cu) and zinc (Zn). TS, VS, TKN, N-NH_4_^+^ and TP were analyzed according to the Standard Methods for the Examination of Water and Wastewater [33]. pH and electrical conductivity were determined using specific electrodes provided by HACH (Derio, Spain). The contents of K were determined by atomic absorption spectroscopy (AAS) (Agilent, Madrid, Spain) preceded by acid extraction, whereas Cu and Zn were analyzed by atomic emission spectroscopy with inductively coupled plasma (ICP-OES) also preceded by acid extraction. All parameters were determined in triplicate.

The soil used for the pot-test was characterized in terms of pH, EC, Olsen phosphorous (Olsen P), exchangeable K, organic matter (OM), calcium (Ca), magnesium (Mg) and sodium (Na). pH and electrical conductivity were determined using specific electrodes provided by HACH preceded by an extraction with water of 1:2.5 and 1:5, respectively. Olsen P was determined by UV-Vis spectrophotometry, whereas OM was determined by potentiometric titration. Exchangeable K, Ca, Mg and Na were analyzed by ICP-OES preceded by extraction with ammonium acetate.

### 2.5. Seedling Growth Tests

Seeding growth tests were used to determine the maximum application rates of products (either VSEP concentrate from pig slurry, PS-concentrate; or VSEP concentrate from digestate, D-concentrate) that can be applied without significant inhibitory effect on the percentage of germinated seeds. The crops selected for carrying out germination inhibition tests were barley (*Hordeum vulgare*) and rapeseed (*Brassica napus*) due to the high relevance of these crops in the study region (Catalonia).

The seeding growth tests were conducted according to the protocols defined by the International Seed Testing Association (ISTA) and the Association of Official Seed Analysts (AOSA). Twenty-five seeds of barley or rapeseed were sown in Petri dishes between 2 filter papers and 3 mL of diluted product (0.5–50%, *v*/*v*) were added. In this study, the range of concentrations for each concentrated product evaluated were 8.0, 11.0, 16.0, 22.0 and 31.0 percent (*v*/*v*) for the tests conducted with barley and 1.75, 3.0, 5.0, 8.0, and 12.5 percent (*v*/*v*) for the tests carried out with rapeseed. These dosages were selected considering the results obtained in range-finding inhibition tests carried out in the range 0.5–50% (*v*/*v*). Each concentration of the products was evaluated in quadruplicate with 25 seeds per replicate, for a representative total number of seeds (100). For the duration of the tests (6–7 d) the Petri dishes were maintained at room temperature (20–22 °C) with diffused light, and the moisture content of each Petri dish was checked daily. The data set obtained in the germination tests was analyzed based on dose–response curves using the *drc* package within version 4.1.3 of the R statistical analysis software.

### 2.6. Pot-Test

The potential agronomic performance of PS-concentrate and D-concentrate was assessed based on the N-criteria (nitrogen contents of the products), with reference negative (no fertilization) and positive controls (fertilization with commercial ammonium sulphate (NH_4_)_2_SO_4_). The three different fertilization treatments were applied at application rates of 30%, 60% and 100% of the N maximum application (170 kg ha^−1^ y^−1^). All fertilizer was applied at the same time as seed sowing. Each pot was considered a replicate, and each treatment had 4 replicates (40 pots in total).

Healthy barley seeds were sown in 2 L capacity pots (16 cm in upper diameter, 10 cm in bottom diameter and 15 cm high). In each pot, 8 seeds were sown in 1.94 kg of a <5 mm sieved loamy soil (13% humidity). Soil characterization is summarized in Table 1. 

Plants were grown in a greenhouse for 90 days, from 22 March to 21 June 2021. Over this period, minimum temperatures increased from 6 °C to 16 °C, and maximum temperatures increased from 16 °C to 27 °C. Plants were irrigated every 2 days and spike development began at day 53 (14 May 2021). Plants were harvested at day 91 (21 Jun 2021). Figure S3 represents the different plant growth stages (sowing, germination, growth and harvesting) during the development of the pot-test.

Plant growth in response to the different products and fertilization rates were assessed in terms of plant height and dry weight. Dry weight was obtained by drying biomass in an oven at 60 °C for 48 h.

The data set obtained in pot tests was statistically analyzed using the IBM SPSS^®^ 28 program (IBM SPSS Statistics, Corporation, Chicago, IL, USA). Analysis of variance (one-factor ANOVA) was conducted, and the Duncan test was applied with a *p* ≤ 0.05, to establish significant differences between treatments.

## 3. Results and Discussion

### 3.1. Feedstock Characterization

The physicochemical characterization of the selected feedstocks used during the three batch tests conducted is summarized in Table 2. The liquid fraction from pig slurry was characterized by an average pH around 8.0 and an electrical conductivity of around 17.2 mS cm^−1^. Variability in the total solids contents was observed between the different sampling campaigns (from 1.8 to 4.2% TS). This feedstock showed a significant content in nutrients: 2.8–3.8 g TKN L^−1^ (around 75% as N-NH_4_^+^), 622–750 mg TP L^−1^ and 1.5–1.6 g K L^−1^. On the other hand, the liquid fraction from the digestate was characterized by an average pH of around 8.7, an electrical conductivity of around 28.8 mS cm^−1^ and a total solid content of around 2.2%. In this case, the nutrient content was very similar between the different sampling campaigns conducted: around 6.2 g TKN L^−1^ (up to 84% as N-NH_4_^+^), 365 mg TP L^−1^ and 494 mg K L^−1^.

### 3.2. Determination of the Optimal Operating Pressure

**Figure 2:**
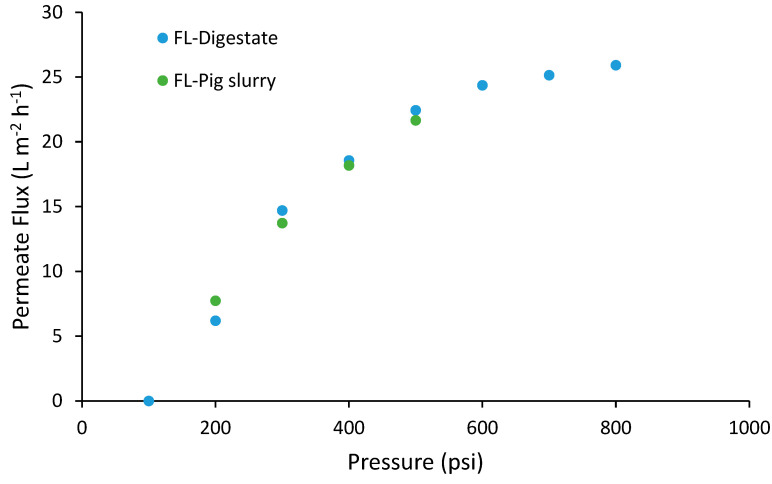
Determination of the optimal operating pressure for the treatment of each feedstock.

Pressure and temperature are two operating parameters with great influence on the efficiency of the VSEP system, with an increase in the permeate flux when the pressure and/or temperature increase. Although the operating temperature was defined by the origin of the feedstock (the liquid fraction from pig slurry was around 20 °C and the digestate around 35 °C), the optimal operating pressure needs to be determined for each feedstock since this parameter is conditioned by its composition. The procedure followed to determine the optimal pressure for each feedstock was: (i) line out at 300 psi in order to stabilize the permeate flow rate, (ii) increasing the working pressure from 200 to 800 psi evaluating the increase in the permeate flow rate and (iii) line out at the working pressure (selected based on the results obtained in stage (ii)) to verify the stabilization of the permeate flow rate. The results obtained for each feedstock are shown in Figure 2.

The optimal operating pressures for the treatment of the LF-digestate and the LF-pig slurry were 500 and 400 psi, respectively. For the LF-digestate, an increase in pressure higher than 500 psi did not have any significant effect on the permeate flow rate. In the case of the LF-pig slurry, the optimal working pressure was selected taking into account that the VSEP system needs a minimum recirculating flow rate for the pump and this recirculating flow rate was 0 at working pressures higher than 400 psi. 

### 3.3. Performance of Water Recovery 

**Figure 3:**
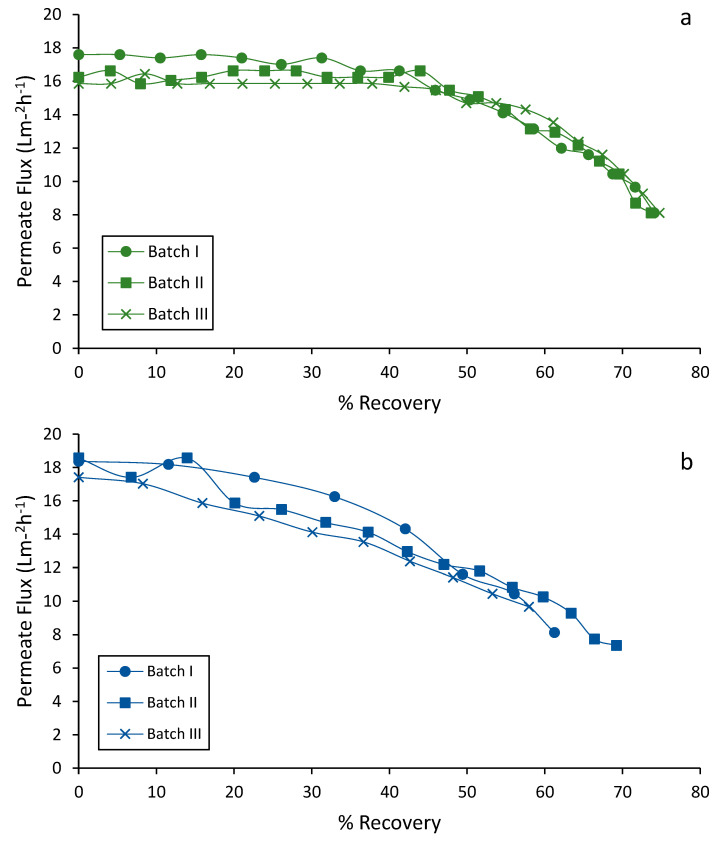
The permeate flux as a function of the permeate recovery for the LF- pig slurry (**a**) and LF-digestate (**b**) tested in the screening study.

Figure 3 shows the permeate flux as a function of the permeate recovery for the LF-pig slurry (a) and LF-digestate (b) tested in the screening study. Both feedstocks show a decline in the permeate flux as the total permeate recovery increases, i.e., the total permeate achieved in each batch. However, the permeate flux for the LF-pig slurry was kept constant up to recovery rates of 40%, whereas the loss of permeate flux for the LF-digestate was observed from the beginning of each batch. Flux decline in membrane filtration is a result of the resistance caused by cake layer formation, as it reduces the effective transmembrane pressure across the membrane, resulting in a lower permeate flux [13]. Thus, the high conductivity of the LF-digestate would lead to early clogging of the membrane resulting in a more abrupt loss of permeate flux, and thus an earlier cleaning would be required.

**Figure 4:**
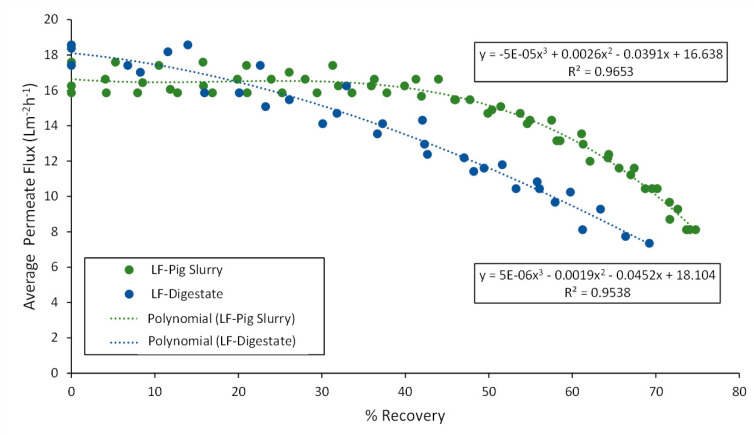
Average permeate flux as a function of permeate recovery for the LF-pig slurry and LF-digestate tested in the screening study.

The average flux and water recovery of the LF-pig slurry were 14.47 Lm^−2^h^−1^ at 76.7% overall permeate recovery operating at 400 psi and 35 °C. Lower values were obtained in the treatment of the LF-digestate, reaching an average flux of 13.72 Lm^−2^h^−1^ at 67.13% overall permeate recovery at 500 psi and 20 °C. The high value in conductivity attributed to the high salt content of the digestate would decrease the recovery of permeate caused by rapid fouling of the membranes. This fact is evidenced by the rapid and progressive loss of permeate flow in each batch carried out with the liquid fraction of the digestate. Thus, the repeatability of the results obtained in each of the experiments allowed a polynomial fit to describe the relationship between permeate flux and permeate recovery (Figure 4). 

### 3.4. Performance of Nutrient Recovery

**Figure 5:**
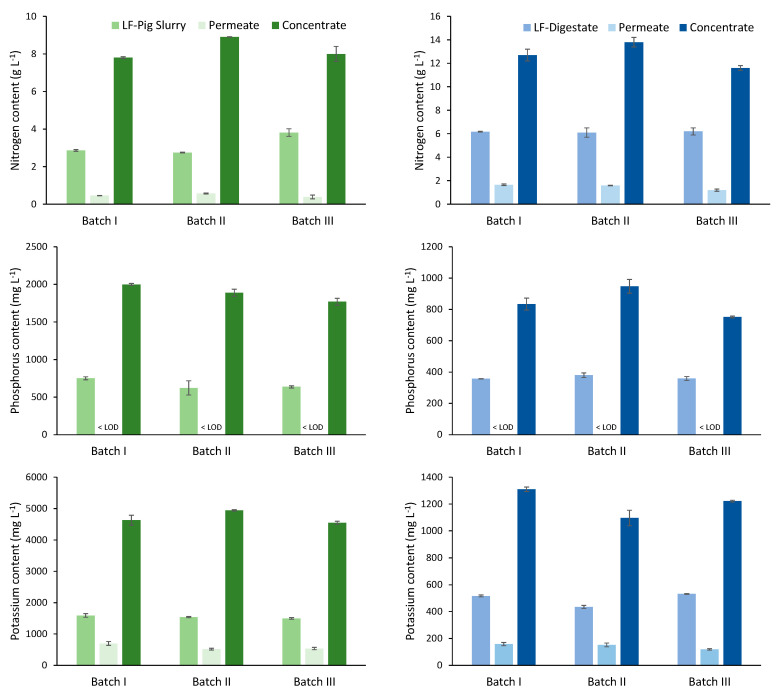
Concentrations of nitrogen, phosphorous and potassium detected in the feedstocks (LF-pig slurry and LF-digestate), permeates and concentrates obtained in each batch test.

Figure 5 shows the NPK content of each feedstock (LF-pig slurry and LF-digestate) and the permeates and concentrates obtained during each batch test using the VSEP technology. The application of the VSEP system for the treatment of the liquid fraction from pig slurry showed high reproducibility during the three batch tests carried out, regardless of the physicochemical characteristics of the feedstock (Table S1—Supplementary material). The high rejection rates observed for nitrogen, phosphorous and potassium (79.3–89.7%, > 99.9% and 56.1–66.6%, respectively) allowed a high-quality PS-permeate with a low content in total solids (<0.01%) and nutrients (0.39–0.57 g TKN L^−1^, < 0.5 mg TP L^−1^ and 514–698 mg K L^−1^) to be obtained. The PS-concentrate obtained was characterized by a high content of nitrogen (7.8–8.9 g TKN L^−1^), phosphorous (1.8–2.0 g TP L^−1^) and potassium (4.9–4.4 g K L^−1^), so this product shows a high potential to be valorized as a bio-based fertilizer in agriculture. The average concentrations of copper and zinc detected in the PS-concentrate (176 and 1061 mg kg^−1^ on dry basis (d.b.), respectively) were below the maximum admissible values (1750 mg Cu/Kg^−1^ d.b. and 4000 mg Zn/Kg^−1^ d.b.) defined in the European regulation (Directive 86/278/EEC) [34], allowing their use as a bio-based fertilizer in agricultural soils. 

During treatment of the LF-digestate at 500 psi, the VSEP technology showed high recovery efficiencies. The main physicochemical parameters of the liquid fraction of digestate are summarized in Table S2-Supplementary Materials. The rejection rates detected for nitrogen (73.5–80.3%), phosphorous (>99.9%) and potassium (65.1–77.7%) allowed a D-permeate with a low total solids content (<0.01%) and nutrient concentrations of 1.7–2.3 g TKN L^−1^, <0.5 mg TP L^−1^ and 119–158 mg K L^−1^ to be obtained. The D-concentrate obtained was characterized by high contents of nitrogen (11.6–13.8 g TKN L^−1^), phosphorous (0.7–0.9 g TP L^−1^) and potassium (1.1–1.3 g K L^−1^). The average metal contents of the concentrate were 210 mg kg^−1^ d.b. for copper and 1006 mg kg^−1^ d.b. for zinc, both values being below the maximum admissible values allowed for application of sludge in agricultural soils (Directive 86/278/EEC). 

In terms of concentration performance, the concentration ratio was estimated and defined as the ratio between the nutrient concentration in the concentrate product and the nutrient concentration in the feedstock [35]. The application of the VSEP technology for the treatment of the liquid fraction of pig slurry resulted in NPK concentration factors of 2.1–3.2, 2.6–3.0 and 2.9–3.2, respectively. These NPK concentration factors were lower for the liquid fraction of the digestate treatment, reaching values of 1.8–2.2, 2.1–2.4 and 2.2–2.5, respectively. Based on other studies testing VSEP technology, nutrient concentration factors were calculated for digestate treatment and manure treatment. Vaneeckhaute et al. (2012) [27] assessed the performance of the VSEP system in the removal of macronutrients (N, P, K) from the liquid fraction of digestate. Concentration factors for NPK were estimated at 2.8, 1.8, and 1.9, respectively. Although the content in macronutrients of the digestate in the two studies differs, concentration factors in the same range were achieved (2–3). On the other hand, higher concentration yields were obtained during the treatment of cow manure using vibrating reverse osmosis [36]. NPK concentration factors of around 5 were achieved. It should be highlighted that these improvements were achieved due to the application of very high operating pressures (600 psi) and consequently filtrate recoveries were well above those observed in this study.

### 3.5. Seedling Growth Tests

**Figure 6:**
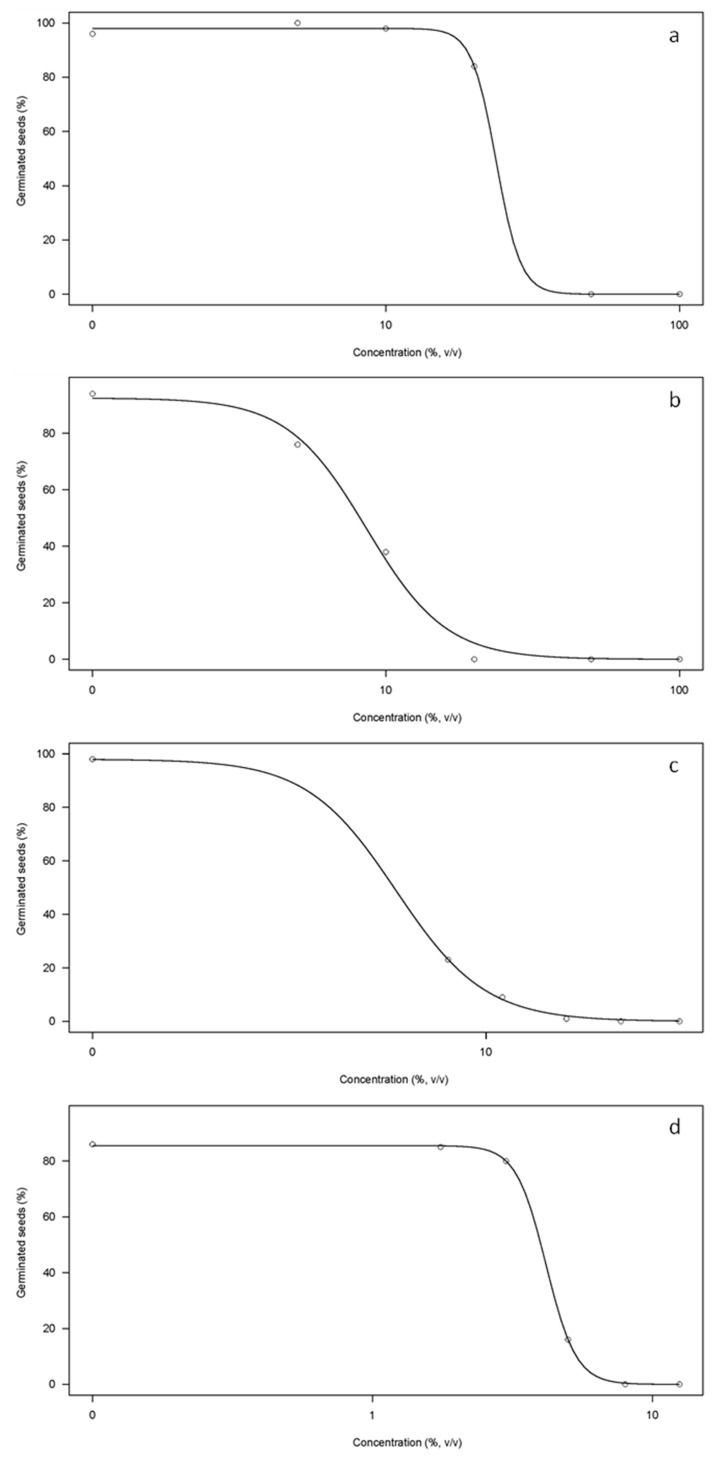
Proportion of germinated seeds for the different concentrations of concentrated products applied: (**a**) barley seeds and PS-concentrate, (**b**) rapeseed and PS-concentrate, (**c**) barley seeds and D-concentrate and (**d**) rapeseed and D-concentrate. The concentrations applied for barley were 0; 8.0; 11.0; 16.0; 22.0; 31.0% (*v*/*v*), whereas for the rapeseed they were 1.75; 3.0; 5.0; 8.0; 12.5% (*v*/*v*).

The results obtained from the germination inhibition tests for barley and rapeseed using either the PS-concentrate or the D-concentrate are shown in Figure 6. In general, an increase in the applied amount of concentrated product resulted in a reduction in the number of germinated seeds. These results are consistent with studies conducted by Munns and Tester (2008) [37] and Rasheed et al. (2014) [38] who evaluated the effect of the salt stress on the growth of barley and rapeseed, respectively.

The obtained results demonstrate a higher inhibitory effect for the D-concentrate compared with the PS-concentrate, especially significant in the case of barley. In this case the germination inhibition of 10% of the barley seeds (EC10) were estimated at 3.3% and 9.4%, for D-concentrate and PS-concentrates, respectively. The higher inhibitory effect of the D-concentrate is likely owing to salinity since the product obtained during the LF-digestate treatment had a higher conductivity than the concentrate obtained during the treatment of the LF-pig slurry (48 mS cm^−1^ vs. 40 mS cm^−1^).

On the other hand, the germination tests have also shown the different potentials in valorization of the concentrate products with respect to crop type. Barley showed a higher resistance than rapeseed to the application of both products as fertilizers, since the EC10 values for barley were 9.4% (PS-concentrate) and 3.3% (D-concentrate) as compared with 3.5% (PS-concentrate) and 3.1% (D-concentrate) for rapeseed. These results are consistent with the literature since barley is identified as the most salt-tolerant cereal crop with a salinity tolerance up to 260 mM NaCl [37].

Although the application of the VSEP technology had already been evaluated for the treatment of digestate and pig slurry liquid fractions [26,28], limited studies were found in the literature assessing the agronomic value of the VSEP-concentrated products on plant growth. Consequently, the germination tests conducted in this study are considered a first step not only to demonstrate the efficiency of the VSEP system to recover nutrients, but also the agronomic value of the products obtained.

### 3.6. Pot-Test

**Figure 7:**
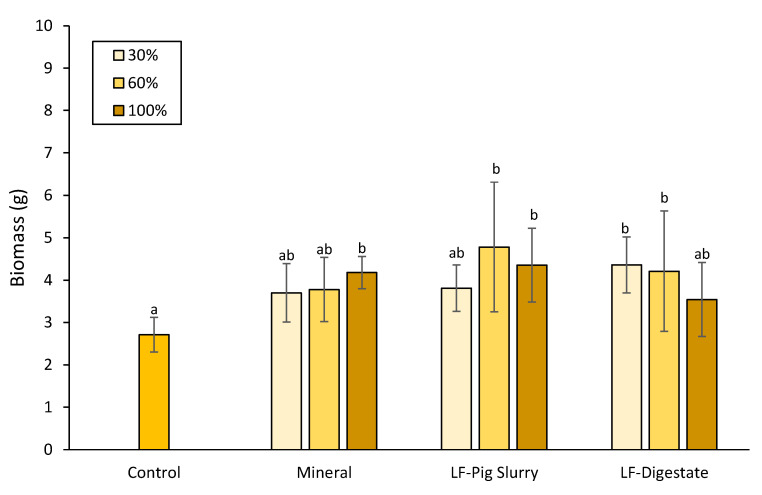
Dry weight (g) of barley plants under different treatments and applied application rates (30%, 60% and 100% of maximum permitted N application). Control, no fertilizer applied to plants. Each column represents the mean values ± SD. Columns with different letters were significantly different according to Duncan’s test (*p* ≤ 0.05).

The highest fertilization application rate (100%) of PS-concentrate and mineral fertilizer significantly increased the total yield in comparison with the negative control. In addition, 60% of the nitrogen application with PS-concentrate showed significant differences as compared with the negative control (Figure 7). These results indicated that the use of PS-concentrate could replace mineral fertilizers, which is consistent with previous works that have tested the mineral fertilizer replacement value of the concentrated liquid fraction of pig slurry, concluding that, under certain conditions and soil types, they have a similar N fertilizer value [39,40].

Regarding the D-concentrate application, the results showed that 30% and 60% of the permitted N fertilization rate resulted in a higher biomass compared with the negative control and were similar to the mineral fertilization. The application of D-concentrate at 100% N slightly decreased plant biomass in comparison with 30% and 60%, although this difference was not statistically significant. Previous literature has shown that the application of digestate to barley can improve crop yields [41,42]. It has also been reported that digestate performed better in terms of barley grain yield than the untreated manure application [43]. However, in this study, the electrical conductivity of the D-concentrate was much higher than the digestates from the literature, and high salinity can impair crop growth [41,43]. Therefore, it may be the case that despite the high salinity tolerance of barley, high application rates of the D-concentrate negatively affected crop growth, as evidenced in other studies [37]. Moreover, the mineral nitrogen forms in the D-concentrate are immediately available to the plant, but this is also associated with rapid losses in the form of ammonia volatilization, reducing the fertilizing capacity of the digestate [44].

## 4. Conclusions and Future Perspective

The efficiency of the vibrating reverse osmosis unit to recover nutrients from the liquid fractions from pig slurry and digestate was demonstrated at pilot scale. The application of the VSEP technology allowed reductions in the water contents of the feedstocks of up to 77%, which translates to decreased costs associated with the transport of livestock waste to other geographical regions with nutrient-deficient soils.

Concentrated products obtained with the VSEP unit were characterized by high contents of nitrogen, phosphorous and potassium, evidencing their potential use as fertilizing products in agriculture. Although both VSEP-concentrated products were characterized by significant nutrient contents and showed an agronomic value similar to mineral fertilizer in barley cultivation, the salinity of the D-concentrate was identified as a key factor which may limit high application rates.

Although this study can be considered as a first step to validate the application of the VSEP system, the results obtained demonstrate the potential of the VSEP technology to be implemented as an alternative technology for nutrient recovery from livestock waste. However, limitations associated with assessment using batch experiments rather than continuous operating conditions require further research in order to implement the technology at full scale.

## Figures and Tables

**Table 1:** Characterization of the soil used for the pot-test.

pH	8.5
EC (dS m^−1^)	0.12
N-NO_3_^−^ (mg kg^−1^ d.b.)	8.7
Olsen P (mg kg^−1^ d.b.)	11.4
Exchangeable K (mg kg^−1^ d.b.)	90.1
Organic matter (mg kg^−1^ d.b.)	1.3
Ca (mg kg^−1^ d.b.)	5289
Mg (mg kg^−1^ d.b.)	82
Na (mg kg^−1^ d.b.)	15

d.b.: dry matter basis.

**Table 2:** Physicochemical characterization of the feedstocks (liquid fractions from pig slurry and digestate).

	LF-Pig slurry			LF-Digestate		
	Batch I	Batch II	Batch III	Batch I	Batch II	Batch III
**pH**	8.23 ± 0.02	8.19 ± 0.02	8.07 ± 0.03	8.86 ± 0.06	8.73 ± 0.03	8.72 ± 0.08
**EC (mS cm^−1^)**	17.7 ± 0.1	16.8 ± 0.1	16.85 ± 0.06	28.8 ± 0.4	28.7 ± 0.7	28.8 ± 0.2
**TS (%)**	2.18 ± 0.01	1.8 ±0.1	4.16 ± 0.06	2.15 ± 0.01	2.2 ± 0.2	2.20 ± 0.06
**VS (% db)**	53.2 ± 0.2	52.3 ± 0.5	54.0 ± 0.1	70.6 ± 0.2	70.8 ± 0.1	70.6 ± 0.6
**N-NH4^+^ (g L^−1^)**	2.2 ± 0.1	2.26 ± 0.03	2.71 ± 0.05	3.03 ± 0.05	5.13 ± 0.08	4.99 ± 0.08
**TKN (g L^−1^)**	2.86 ± 0.05	2.75 ± 0.01	3.81 ± 0.05	6.18 ± 0.03	6.1 ± 0.4	6.2 ± 0.3
**P (mg L^−1^)**	750 ± 20	622 ± 95	636 ± 14	357 ± 1	380 ± 15	359 ± 12
**K (mg L^−1^)**	1591 ± 59	1538 ± 20	1497 ± 26	517 ± 7	435 ± 11	532 ± 3

## Data Availability

The data presented in this study are available on request from the corresponding author.

## Supplementary Materials

The following supporting information can be downloaded at: https://www.mdpi.com/article/10.3390/membranes12090848/s1. Figure S1. VSEP unit applied at pilot scale for the treatment of livestock waste; Figure S2. Filtration module cross section of the VSEP unit applied at pilot scale; Figure S3. Evolution of the plants during the development of the pot-test: (A) seeding, (B) germination, (C) growth and D) harvesting; Table S1. Physicochemical characterisation of the products obtained (permeate of pig slurry (PS-P) and concentrate of pig slurry (PS-C)) during the treatment of the liquid fraction of pig slurry (PS) with VSEP technology; Table S2. Physicochemical characterisation of the products obtained (permeate of digestate (D-P) and concentrate of digestate (D-C)) during the treatment of the digestate (D) with VSEP technology.

## Abbreviations

AAS—Atomic Absorption Spectroscopy; AOSA—Association of Official Seed Analysts; Ca—Calcium; Cu—Copper; D—Digestate; d.b.—Dry matter basis; EC—Electrical conductivity; ICP-OES—Atomic Emission Spectroscopy with Inductively Coupled Plasma; ISTA—International Seed Testing Association; K—Potassium; LF—Liquid fraction; N—Nitrogen; Na, Sodium; Mg—Magnesium; OM—Organic matter; P—Phosphorous; PS—Pig slurry; TKN—Total Kjeldahl nitrogen; TP—Total phosphorous; TS—Total solids; VS—Volatile solids; VSEP—Vibratory Shear Enhanced Processing, Zn—Zinc.
