# Supplementary material for: Application of Vibrating Reverse Osmosis Technology for Nutrient Recovery from Pig Slurry in a Circular Economy Model
Source: Membranes (Basel). 2022 Aug 30;12(9):848. doi: 10.3390/membranes12090848 (PMC9501425; doi:10.3390/membranes12090848)
Supplement: Supplementary file 1 [file membranes-12-00848-s001.zip › membranes-1866428-supplementary.pdf]

membranes

MDPI

Supplementary material

Application of vibrating reverse osmosis technology for nutrient recovery from pig slurry in a circular economy model

Esther Vega *†, Lidia Paredes †, Evan A.N. Marks, Berta Singla, Omar Castaño-Sánchez, Carme Casas, Rosa Vilaplana, Mabel Mora, Sergio Ponsá and Laia Llenas

BETA Technological Center, TECNIO Network, University of Vic—Central University of Catalonia, C/de Roda 70, 08500 Vic, Spain
* Corresponding author: esther.vega@uvic.cat
† Equally contributed to the work.

Citation: Vega, E.; Paredes, L.; Marks A.N. Evan; Singla, B.; Castaño-Sánchez, O.; Casas, C.; Vilaplana, R.; Mora, M.; Ponsá, S.; Llenas, L. Application of vibrating reverse osmosis technology for nutrient recovery from pig slurry in a circular economy model. *Membranes* **2022**, *12*, 848. https://doi.org/10.3390/membranes12090848

Academic Editor(s): Xanel Vecino

Received: 29 July 2022
Accepted: 25 August 2022
Published: 30 August 2022

Publisher's Note: MDPI stays neutral with regard to jurisdictional claims in published maps and institutional affiliations.

Copyright: © 2022 by the authors. Licensee MDPI, Basel, Switzerland. This article is an open access article distributed under the terms and conditions of the Creative Commons Attribution (CC BY) license (https://creativecommons.org/licenses/by/4.0/).

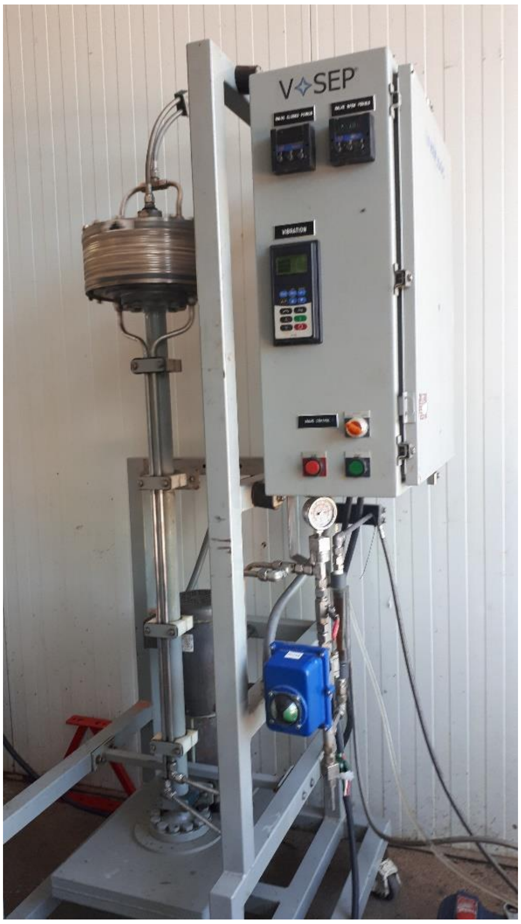

Figure S1. VSEP unit applied at pilot scale for the treatment of livestock waste.

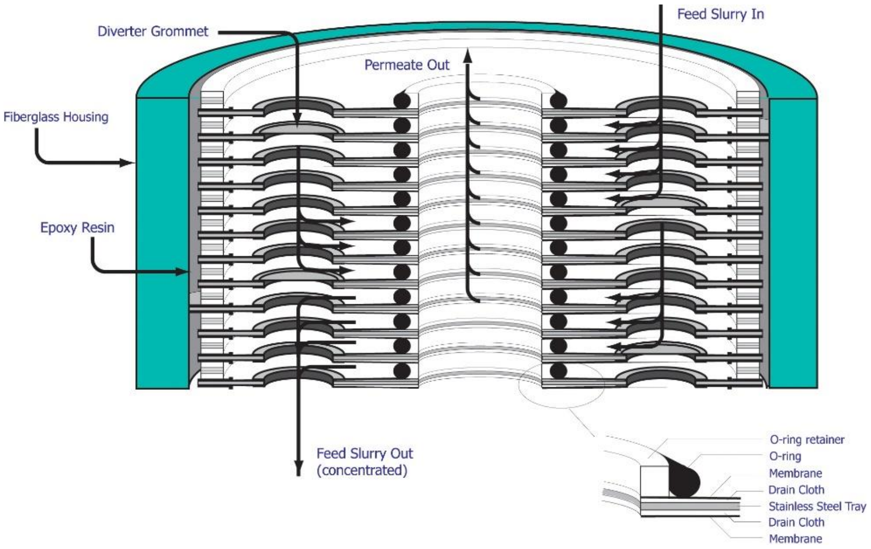

Figure S2. Filtration module cross section of the VSEP unit applied at pilot scale.

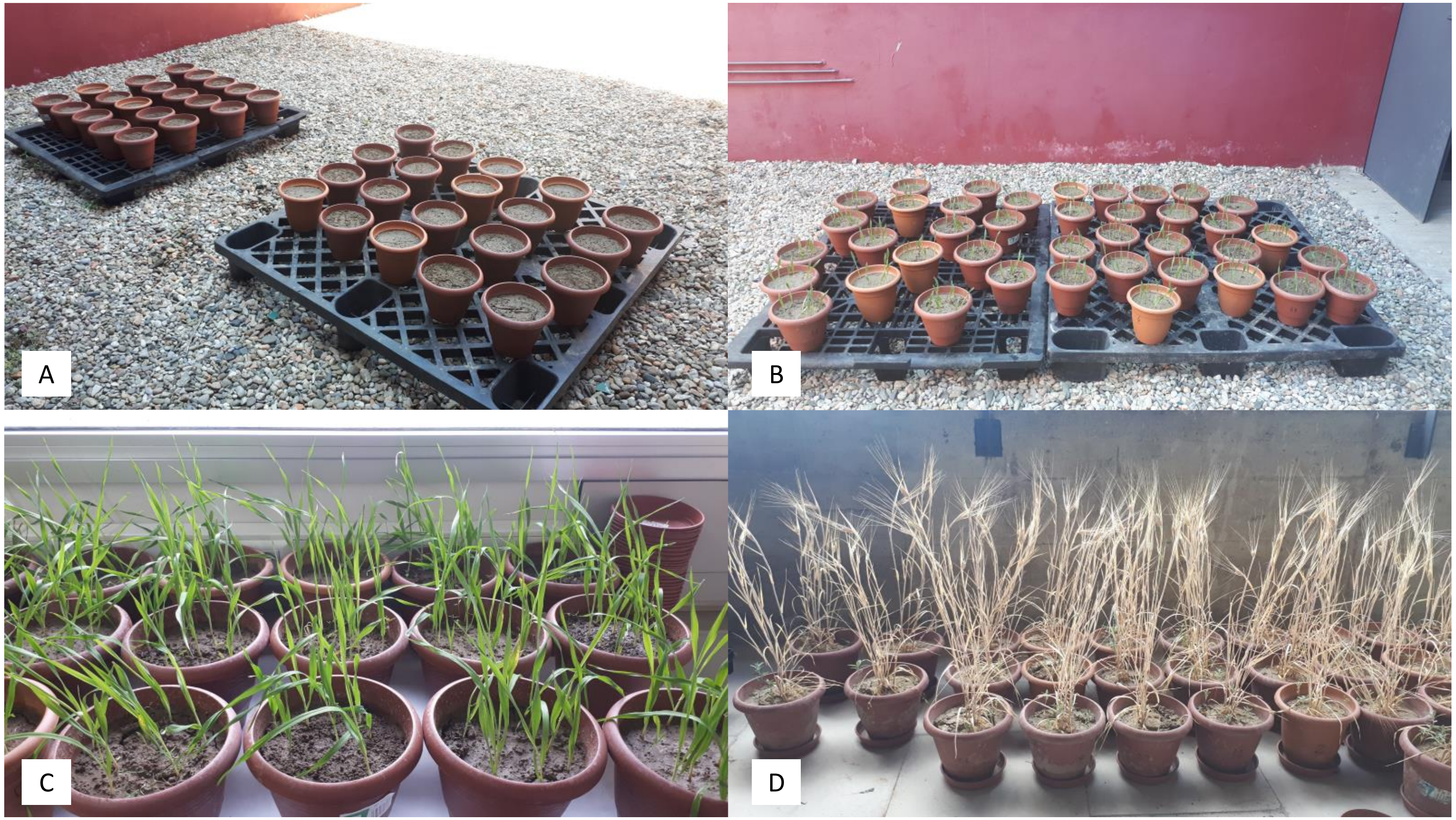

Figure S3. Evolution of the plants during the development of the pot-test: A) seeding, B) germination, C) growth and D) harvesting.

Table S1. Physicochemical characterisation of the products obtained (permeate of pig slurry (PS-P) and concentrate of pig slurry (PS-C)) during the treatment of the liquid fraction of pig slurry (PS) with VSEP technology.

	**Batch I**			**Batch II**			**Batch III**		
	**PS**	**PS-P**	**PS-C**	**PS**	**PS-P**	**PS-C**	**PS**	**PS-P**	**PS-C**
**pH**	8.23 ± 0.02	9.71 ± 0.01	8.72 ± 0.01	8.19 ± 0.02	9.61 ± 0.01	8.82 ± 0.01	8.07 ± 0.03	9.66 ± 0.04	8.70 ± 0.02
**λ (mS cm^{-1})**	17.7 ± 0.1	3.05 ± 0.06	40.2 ± 0.5	16.8 ± 0.1	2.85 ± 0.06	39.7 ± 0.2	16.85 ± 0.06	2.0 ± 0.0	40.1 ± 0.1
**TS (%)**	2.18 ± 0.01	< 0.01	7.04 ± 0.04	1.8 ±0.1	< 0.01	6.91 ± 0.01	4.16 ± 0.06	< 0.01	6.13 ± 0.01
**VS (%)**	53.2 ± 0.2	< 0.01	54.9 ± 0.1	52.3 ± 0.5	< 0.01	54.1 ± 0.1	54.0 ± 0.1	< 0.01	53.7 ± 0.1
**N-NH4^{+} (g L^{-1})**	2.2 ± 0.1	0.47 ± 0.03	6.1 ± 0.2	2.26 ± 0.03	0.56 ± 0.05	6.1 ± 0.1	2.71 ± 0.05	0.34 ± 0.03	6.0 ± 0.2
**TKN (g L^{-1})**	2.86 ± 0.05	0.46 ± 0.02	7.8 ± 0.2	2.75 ± 0.01	0.57 ± 0.02	8.9 ± 0.1	3.81 ± 0.05	0.39 ± 0.01	8.0 ± 0.4
**P (mg L^{-1})**	750 ± 20	< 0.5	1998 ± 13	622 ± 95	< 0.5	1888 ± 47	636 ± 14	< 0.5	1769 ± 47
**K (mg L^{-1})**	1591 ± 59	698 ± 62	4627 ± 159	1538 ± 20	514 ± 27	4941 ± 22	1497 ± 26	535 ± 38	4545 ± 50

* The physicochemical characterisation of the liquid fraction corresponds to the pre-filtered waste (SWECO, 120 μm)

Table S2. Physicochemical characterisation of the products obtained (permeate of digestate (D-P) and concentrate of digestate (D-C)) during the treatment of the digestate (D) with VSEP technology.

	**Batch I**			**Batch II**			**Batch III**		
	**D**	**D-P**	**D-C**	**D**	**D-P**	**D-C**	**D**	**D-P**	**D-C**
**pH**	8.86 ± 0.06	9.76 ± 0.06	8.90 ± 0.06	8.73 ± 0.03	10.0 ± 0.2	8.89 ± 0.03	8.72 ± 0.08	9.85 ± 0.06	8.79 ± 0.06
**λ (mS cm^{-1})**	28.8 ± 0.4	6.8 ± 0.2	48.7 ± 0.1	28.7 ± 0.7	8.6 ± 0.1	48.7 ± 0.8	28.8 ± 0.2	5.8 ± 0.1	46.8 ± 0.1
**TS (%)**	2.15 ± 0.01	< 0.01	4.77 ± 0.01	2.2 ± 0.2	< 0.01	5.67 ± 0.01	2.20 ± 0.06	< 0.01	4.46 ± 0.01
**VS (%)**	70.6 ± 0.2	< 0.01	72.3 ± 0.1	70.8 ± 0.1	< 0.01	72.1 ± 0.1	70.6 ± 0.6	< 0.01	71.4 ± 0.1
**N-NH4^{+} (g L^{-1})**	3.03 ± 0.05	1.64 ± 0.02	9.9 ± 0.1	5.13 ± 0.08	1.55 ± 0.02	10.4 ± 0.1	4.99 ± 0.08	1.2 ± 0.1	8.8 ± 0.1
**TKN (g L^{-1})**	6.18 ± 0.03	1.66 ± 0.06	12.7 ± 0.5	6.1 ± 0.4	1.60 ± 0.02	13.8 ± 0.4	6.2 ± 0.3	1.2 ± 0.1	11.6 ± 0.2
**P (mg L^{-1})**	357 ± 1	< 0.5	834 ± 38	380 ± 15	< 0.5	947 ± 44	359 ± 12	< 0.5	752 ± 6
**K (mg L^{-1})**	517 ± 7	158 ± 12	1310 ± 17	435 ± 11	152 ± 14	1096 ± 58	532 ± 3	119 ± 6	1222 ± 6

* The physicochemical characterisation of the digestated corresponds to the pre-filtered waste (CENTRIFUGE + SWECO, 120 μm)
